# A Cross-Sectional Investigation of the Association between Arterial Stiffness and Depressive Symptoms, Anxiety Symptoms, and Quality of Life

**DOI:** 10.3390/medicina59030477

**Published:** 2023-02-28

**Authors:** Tae-Kyung Yoo, Seunghee Lee, Soo-Young Hwang, Curie Ahn, Saejong Park, Jong-Young Lee

**Affiliations:** 1 Department of Medicine, MetroWest Medical Center, Framingham, MA 01702, USA; 2 Department of Physical Education, Korea University, Seoul 02841, Republic of Korea; 3 Harvard T.H. Chan School of Public Health, Boston, MA 02115, USA; 4 Division of Sports Science, Korea Institute of Sport Science, Seoul 01794, Republic of Korea; 5 Division of Cardiology, Department of Internal Medicine, Kangbuk Samsung Hospital, Sungkyunkwan University School of Medicine, Seoul 03181, Republic of Korea

**Keywords:** arterial stiffness, depressive symptoms, anxiety symptoms, quality of life

## Abstract

*Background and Objectives*: Previous studies have assessed the association between arterial stiffness and depressive and anxiety symptoms, but the results were inconsistent. We aimed to conduct a cross-sectional study to assess the relationship between arterial stiffness, depressive and anxiety symptoms, and quality of life. *Materials and Methods*: We analyzed the 2014–2015 Korea Institute of Sport Science Fitness Standards project data. Brachial-ankle pulse wave velocity (baPWV) was measured to assess arterial stiffness. High baPWV was defined as a baPWV higher than 1400 cm/s. Participants completed Beck’s depressive symptoms inventory (BDI), Beck’s anxiety symptoms inventory (BAI), and the World Health Organization’s Quality of Life Questionnaire (WHOQOL-Bref). We performed a logistic regression analysis by adjusting confounding factors and used the inverse probability of treatment weighting (IPTW) method. *Results*: 1936 participants were included in the analysis (men 43.9%, median age 47). Participants with a high baPWV had higher odds of depressive symptoms compared to those with a normal baPWV (aOR 1.920, 95% CI 1.062–3.472, *p* = 0.031; IPTW OR 2.637, 95% CI 1.219–5.704, *p* = 0.014). In addition, baPWV was significantly associated with depressive symptoms in the IPTW model in men but not in women (OR 2.497, 95% CI 1.004–6.207, *p* = 0.049). High baPWV was not associated with anxiety symptoms in all models, but it was associated with poor QOL in women (OR 4.561, 95% CI 1.465–14.199, *p* = 0.009). *Conclusions*: High baPWV was associated with higher odds of depressive symptoms, especially in men. Our study suggests a modest association between arterial stiffness and depressive symptoms in Korean adults.

## 1. Introduction

Mental health disorders are a prevalent condition, causing a significant burden worldwide [1,2]. Approximately 1% of the world’s population currently lives with a mental disorder [1,2]. Among the mental health disorders, depression and anxiety disorders are the leading contributors to this burden [3]. In addition, depression and anxiety disorders significantly diminish the quality of life of affected individuals [4]. While a mental disorder in itself has a negative effect on each individual’s life, it also causes many comorbidities [5]. The sympathetic response is increased in patients with a mental health disorder, and their negative impact on cardiovascular disease has been reported [5]. This impact contributes to the early mortality of patients with mental disorders [5].

Arterial stiffness is a measure of the elasticity of the blood vessel wall and a marker of atherosclerotic cardiovascular disease in high-risk and healthy community-dwelling individuals [6]. Multiple studies have tried to assess the association between arterial stiffness and mental health disorders [7,8,9,10]. For example, persistent depressive symptoms were associated with the progression of arteriosclerosis as measured by the brachial-ankle pulse wave velocity (baPWV) in Japanese men [11]. The study, using the cohort of the Maastricht Study, also showed the cross-sectional association between arterial stiffness and depression in middle-aged men [10]. In addition, anxiety symptoms were associated with decreased arterial compliance [12]. However, controversy exists as the Health, Aging, and Body Composition Study failed to show an association between depressive symptoms and arterial stiffness. In addition, many studies reported inconsistent relationships between baPWV and depressive symptoms in both men and women [13].

To clarify this gap in clinical knowledge, we wanted to assess the relationship between arterial stiffness and depressive and anxiety symptoms and quality of life in a single study cohort, as most previous studies have evaluated these associations in separate cohorts [12,13]. Therefore, we conducted a cross-sectional study to assess the relationship between arterial stiffness, expressed as baPWV, and depressive symptoms, anxiety symptoms, and the quality of life of study participants.

## 2. Materials and Methods

### 2.1. Study Population

We analyzed the 2014–2015 Korea Institute of Sport Science Fitness Standards (KISS FitS) project data. The details of the cohort and study methods have been described in previous studies by our team [1,14]. Briefly, the KISS FitS project recruited participants among Korean adults aged 19 to 64. All the participants agreed to a health examination and physical fitness level assessment at health examination centers in Republic of Korea. Participants had multiple tests, including a laboratory analysis and anthropometric measurements. Their health-related behavior and medical history were collected through self-reported, standardized questionnaires. Participants with leg claudication were excluded during the recruitment process, as baPWV is known to be less accurate in patients with peripheral arterial disease [15]. The baPWV was measured in a standardized manner using a VP-1000 (Colin Co., Komak, Japan) device [1,14]. The Korea Institute of Sport Science Research Ethics Committee approved the study (No. KISS-201504-EFS-001-01). Participants who agreed to participate in the study provided informed consent during recruitment.

**Figure 1:**
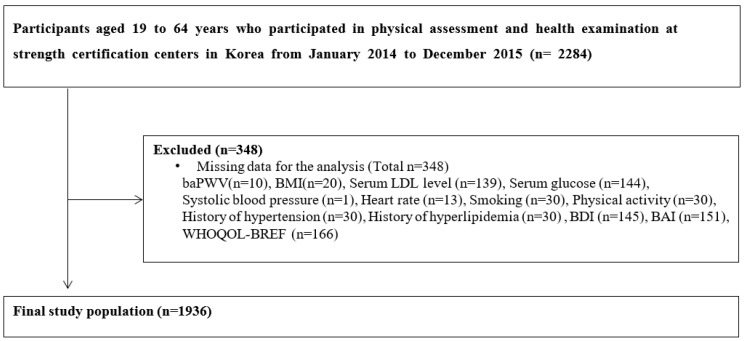
Flow diagram of study participants. Abbreviations: baPWV, brachial-ankle pulse wave velocity; BMI, body mass index; LDL, low density lipoprotein cholesterol; BDI, Beck’s depression inventory; BAI, Beck’s anxiety symptoms index; WHOQOL-Bref, World Health Organization’s Quality of Life Questionnaire.

Among 2284 participants, participants with missing data in the analysis were excluded. A total of 1936 participants were included in the final analysis (Figure 1). After the baPWV measurement, participants were divided into two groups based on their baPWV level: baPWV ≥ 1400 cm/s (high baPWV group) and baPWV < 1400 cm/s (normal baPWV), based on previous reports which have suggested a baPWV cutoff of 1400 cm/s as an independent risk factor in the Framingham score and as a cutoff for distinguishing patients with atherosclerotic cardiovascular disease [16,17].

### 2.2. Assessment of Depressive Symptoms

We used Beck’s depressive symptoms inventory (BDI) to assess patients’ depressive symptoms, validated among the general population in Republic of Korea [18]. The BDI has been used as a screening tool to detect depression in normal populations [19]. The BDI was reported as a test with strong internal consistency, test–retest reliability, and a strong correlation with other depressive symptoms-related self-report measures [18]. The questionnaire consists of a 21-item self-reporting questionnaire to evaluate the severity of depressive symptoms in normal and psychiatric populations [19]. Participants could score from 0 to 3 for each question. A higher sum of scores indicates a more severe presentation of depressive symptoms. The maximum score is 63 and scores equal to or above 20 indicate depressive symptoms (≥20) in non-clinical populations, while further stratification into minimal, mild, moderate, and severe depression has been suggested in those who were diagnosed with depression [19]. As BDI was used as a screening tool in the general population in our study, we defined depressive symptoms as BDI ≥ 20, following previous reports [19,20].

### 2.3. Assessment of Anxiety Symptoms

Beck’s anxiety symptoms inventory (BAI) was used to assess anxiety symptoms [21]. BAI is used frequently in clinical and non-clinical settings. It consists of a 21 self-report questionnaire [22]. For each question, a participant can rate themselves from 0 to 63, with higher scores indicating more severe anxiety symptoms [23]. Its reliability and validity have been previously proven in the Korean population [22,23]. A cutoff score of 16 or greater (≥16) was suggested by the original study and by a meta-analysis of studies reporting cutoff scores for identifying clinically significant anxiety symptoms. In addition, multiple studies have validated the cutoff of 16 for effectively differentiating participants with anxiety symptoms [24,25,26,27,28,29]. Our study defined anxiety symptoms as BAI ≥ 16, following previous reports [24,25].

### 2.4. Assessment of Quality of Life

The World Health Organization’s Quality of Life Questionnaire (WHOQOL-Bref) is used to assess participants’ quality of life [30]. It assesses the quality of life (QOL) in four domains: physical health, psychological, social relationships, and environment [31]. There are 24 items in 4 domains and 2 additional items—an overall QOL and general health (total 26 items) [31]. A higher score indicates a better QOL [31]. A cutoff score of 59 or below (≤59) was defined as poor quality of life, following previous reports [32,33].

### 2.5. Statistical Analysis

The distribution of the variables was tested using the Kolmogorov–Smirnov and Shapiro–Wilk tests. The median and interquartile range were used to express continuous variables with non-normal distributions. Categorical variables were expressed as percentages. The Chi-square test was used to compare the means of categorical variables, and the independent t-test was used for continuous variables. One-way analysis of variance (ANOVA) was used for the trend analysis. Logistic regression was conducted to assess the cross-sectional relationship between the dependent (depressive symptoms, anxiety symptoms, poor quality of life, defined as described above) and independent (high baPWV) variables. Both dependent variables and independent variables were transformed into categorical variables for the analysis (BDI ≥ 20, depressive symptoms; BAI ≥ 16, anxiety symptoms; WHOQOL-Bref ≤ 59, poor quality of life; baPWV ≥ 1400 cm/s, high baPWV). Depressive symptoms, anxiety symptoms, and quality of life were assessed in separate statistical modules (e.g., a module that assessed depressive symptoms did not include anxiety symptoms or quality-of-life variables).

Multiple models were created for the multivariable analysis. Model 1 was adjusted for age and sex. Model 2 was adjusted for anthropometric measurements (systolic blood pressure, heart rate, BMI) in addition to the adjustments in Model 1. Model 3 was adjusted for underlying comorbidities and laboratory values (hypertension, hyperlipidemia, serum LDL, glucose) in addition to the adjustments in Model 2.

Model 4 was adjusted for lifestyle factors, such as smoking and physical activity, in addition to the adjustments in Model 3. Finally, inverse probability of treatment weighting (IPTW) was performed using Model 4. The odds ratio and 95% confidence interval were calculated. Using Model 4 in the logistic regression model, predicted probabilities were calculated to plot the ROC curve. The area under the curve (AUC) of the ROC curve was calculated to estimate the predictive performance of the logistic regression models. A subgroup analysis was conducted in the subgroups (men and women; age ≥ 50 and age < 50). A two-tailed *p*-value of <0.05 was considered statistically significant. All statistical analyses were conducted using IBM SPSS 28.0 (IBM Statistics; IBM Corp., Chicago, IL, USA).

## 3. Results

### 3.1. Baseline Characteristics

Among the 1936 participants, 1589 (82.1%) belonged to the normal baPWV group and 347 participants (17.9%) belonged to the high baPWV group. The high baPWV group participants showed higher median age, men proportion, systolic blood pressure, BMI, LDL, fasting glucose level, hypertension, and hyperlipidemia (*p* = 0.001 for BMI, *p* = 0.023 for hyperlipidemia, all other *p* < 0.001). There was no significant difference in physical activity or smoking rate (Table 1). Depressive symptoms were more common in the high baPWV group (*p* = 0.023, p_trend_ = 0.023). At the same time, there was no significant difference in anxiety symptoms or poor quality of life between the normal baPWV and high baPWV groups (Table 2).

### 3.2. Multivariable Analysis to Assess the Relationship between baPWV and Anxiety Symptoms, Depressive Symptoms, and Poor Quality of Life

High baPWV was significantly associated with depressive symptoms in the crude model (OR 1.707, 95% CI 1.073–2.715, *p* = 0.024). This association remained significant after age and sex adjustment (Model 1, OR 1.675, 95% CI 1.001–2.803, *p* = 0.049). The association remained significant with further adjustment of anthropometric measurement, lab values, and underlying comorbidities (Model 2, OR 2.003, 95% CI 1.120–3.581, *p* = 0.019; Model 3, OR 2.000, 95% CI 1.102–3.628, *p* = 0.023) and additional adjustment of lifestyle factors (Model 4, OR 1.920, 95% CI 1.062–3.472, *p* = 0.031). The AUC of the ROC curve of the logistic regression model for depressive symptoms was 0.907, with 95% CI 0.892–0.922. Lastly, the IPTW model was created using Model 4 and showed a significant association between high baPWV and depressive symptoms (OR 2.637, 95% CI 1.219–5.704, *p* = 0.014).

A high baPWV was not significantly associated with anxiety symptoms in all models and the crude model. However, the adjusted models and the IPTW model showed trends toward a greater odds ratio for patients with high baPWV. In addition, high baPWV was not associated with poor quality of life in all models, including crude, adjusted, and IPTW models (Table 3).

When we divided the participants into men and women, men with a high baPWV showed a significantly higher incidence of depression in the IPTW model (OR 2.497, 95% CI 1.004–6.207, *p* = 0.049). However, there were no significant associations between high baPWV and anxiety symptoms and poor QOL. In female participants, a high baPWV was not associated with depression or anxiety symptoms after the adjustment. High baPWV was significantly associated with poor QOL in women in the IPTW model (OR 4.561, 95% CI 1.465–14.199, *p* = 0.009; Table 4). Lastly, a further analysis was carried out by stratifying participants into age ≥ 50 and age < 50; statistical significance was lost, but there was a non-significant increased trend of association with high baPWV and depressive and anxiety symptoms and poor quality of life in both age ≥ 50 and age < 50 group (Supplemental Table S1).

## 4. Discussion

Our finding showed that the odds of depressive symptoms in those with high baPWV was 1.92 times (95% CI 1.062–3.472; *p* = 0.031) the odds of depressive symptoms in those with normal baPWV after fully adjusting for age, sex, underlying comorbidities, anthropometric measurements, lab values, and lifestyle factors. When we assessed men and women separately, this association was statistically significant in men, while it was not statistically significant in women in the IPTW model. However, there was a trend toward a greater odds ratio in women for depressive symptoms in patients with high baPWV. There was no significant association between arterial stiffness assessed by baPWV levels and anxiety symptoms in both men and women, while arterial stiffness was associated with poor quality of life in women in IPTW model.

The relationship between arterial stiffness and depressive or anxiety symptoms is bidirectional [34]. Arterial stiffness is associated with cerebral small vessel disease, which may predispose individuals with arterial stiffness to depression via the disruption of frontal and subcortical structures involved in mood regulation [35]. Additionally, arterial stiffness is associated with increased sympathetic nerve firing, which is frequently observed in people with depressive and anxiety symptoms [36,37,38]. Individual differences in inflammatory responses to psychophysiological stress are related to changes in artery walls—specifically proven in carotid arteries [39]. On the other hand, the pathophysiology of depression includes increased proinflammatory cytokines [40]. Anxiety is associated with increased inflammation via stress response activation and increased cytokine release from immune cells [41]. Recent studies have emphasized the importance of inflammation in the pathogenesis of arterial stiffness, and ample evidence demonstrates the association of inflammation with arterial stiffness [42]. It is possible that an increased inflammatory response due to depressive or anxiety symptoms might mediate the worsening of arterial stiffness. Furthermore, the ‘Vascular Depressive symptoms’ hypothesis suggests that cerebrovascular disease may predispose to geriatric depressive syndromes with the support of vascular disease and vascular risk factors [43]. This complex relationship, as well as the poor QOL resulting from depressive and anxiety symptoms, has led to multiple studies investigating the association between arterial stiffness and depressive symptoms and anxiety symptoms [4,44]. However, conflicting results have been reported [10].

The Health ABC study performed in 2488 participants showed no relationship between arterial stiffness as measured by carotid-femoral pulse wave velocity (cfPWV) and depressive symptoms as measured by the Center for Epidemiological Studies-Depressive symptoms scale (CES-D) [45]. The study had several limitations, as it did not include the Asian population and did not assess each sex separately [45]. In addition, the study population included only high-functioning African American and White adults aged 70–79 years. Onete et al. showed that greater arterial stiffness, measured by cfPWV, was associated with depressive symptoms—as measured by a Mini-International Neuropsychiatric Interview (MINI) and the Patient Health Questionnaire-9 (PHQ-9)—in men, but not in women [10].

Overall, our study is in line with Onete et al. Aortic stiffness was modestly associated with depressive symptoms, and the association was more prominent in men than in women. Several factors could explain this: First, estrogen plays a protective role in the development of arterial stiffness [46]. The increased estrogen levels of pre-menopausal women could have masked the relationship. Second, though there was no significant relationship, there was a trend toward an increased odds ratio in the women group. It is possible that an association might be revealed in a study with a larger number of participants. Third, women are twice as likely to develop depressive symptoms than men [47]. As the development of depressive symptoms is multifactorial, an alternative explanation for this finding is that other factors contributing to the development of depressive symptoms might have overshadowed the association between arterial stiffness and depressive symptoms in women [10].

The Health ABC study also failed to show a significant relationship between arterial stiffness and anxiety symptoms as measured by the Hopkins Symptom Checklist [45]. A cross-sectional survey of Japanese men was unable to show a significant association between arterial stiffness as measured by baPWV and anxiety symptoms as measured by Profile of Mood States (POMS) [48]. Our results are consistent with prior studies; however, there was a trend of increased odds ratios of high baPWV in participants with anxiety symptoms. Several factors need to be considered in interpreting the result of our study: First, the current study participants consist of people who voluntarily participated in the health examination, including the physical fitness test [1,14]. This suggests our participants are active overall and are less limited by arterial stiffness or anxiety symptoms, which may partially explain why there was no significant association between arterial stiffness and anxiety symptoms [45]. Furthermore, our study participants showed a higher level of physical activity level than that of the average Korean population (50.30% vs. 45.6%) [49]. As physical activity levels are associated with decreased anxiety symptoms, this might have mitigated the potential relationship [50].

As far as the authors know, few studies exist regarding the relationship between arterial stiffness and quality of life. Tap et al. showed an association between arterial stiffness as measured by aortic pulse wave velocity (aPWV) and QOL as measured by the EQ-5D index and visual analog scale in a geriatric population. The study showed that aortic stiffness was associated with poor quality of late life [51]. The authors adjusted for multiple comorbidities and concluded that aortic stiffness is independently associated with QOL. However, the study was limited by a relatively small sample size (n = 280), including only the geriatric population. In addition, though the EQ-5D index is a validated questionnaire, it does not include the social component of quality of life, and it is particularly useful in a rehabilitation setting [51,52]. Using the WHOQOL-Bref form, we were able to assess the social aspect of quality of life [31]. Our results showed a significant relationship between the quality of life and baPWV only in women, but the confidence interval was very wide. Quality of life can be affected by various factors from physical and psychological health to socioeconomic factors such as education, residential area, spouse, and employment [53]. Further studies adjusting for these factors are needed to verify our study results.

Our study is unique in that the cohort represents the general Korean population. In addition, we were able to analyze multiple associations between arterial stiffness and mental disorders in a single cohort. Furthermore, we assessed subjective symptoms and quality of life using validated forms. However, our study also has several limitations: First of all, though BDI and BAI are the validated measurements of depressive and anxiety symptoms for screening purposes, they cannot provide a formal diagnosis of depressive disorder or anxiety disorders [54,55]. Second, our study is a cross-sectional study, and we cannot suggest a causal relationship due to temporality. Third, our study was conducted based on the general Korean population, which is mostly composed of an ethnically homogeneous Asian population. Fourth, our study did not include the geriatric population. Fifth, the number of participants with depressive symptoms was relatively small; however, we mitigated this limitation by performing an IPTW analysis. In addition, the prevalence of depressive symptoms in our study (5.06%) is similar to the prevalence of depressive symptoms in the Korean population (5.3%), suggesting that our study can represent the community [56]. Sixth, the ankle-brachial index was not available in our study data, so we excluded participants with leg claudication during the recruitment process. However, considering the relatively low prevalence of peripheral arterial disease in adults in their 40s in high-income countries, our result was less likely to be affected by peripheral arterial disease [57]. Lastly, other clinical variables including previous comorbidities such as diabetes or dyslipidemia and drug treatments were not available in our study; however, we mitigated this limitation by adjusting for serum glucose and LDL levels.

## 5. Conclusions

In conclusion, the results from this cross-sectional study showed that arterial stiffness is associated with depression in the general population. This result was significant after adjusting for multiple confounding factors such as age, sex, multiple comorbidities, and lifestyle factors. However, arterial stiffness did not significantly correlate with anxiety symptoms and poor quality of life. Future prospective cohort studies incorporating a diverse ethnicity and a broader age group are needed to further examine the relationship between arterial stiffness and depressive symptoms and anxiety symptoms and quality of life.

## Figures and Tables

**Table 1:** Baseline characteristics of the study population.

	Total	Normal baPWV	High baPWV	*p* Value
Number of participants	1936 (100)	1589 (82.1)	347 (17.9)	
baPWV	1201.50 (1078.13–1348.38)	1157.0 (1058–1264.75)	1514.5 (1445–1608.5)	<0.001
Age	47 (35–56)	44 (33–54)	56 (51–61)	<0.001
Men	885 (45.7)	697 (43.9)	188 (54.17)	<0.001
Systolic blood pressure (mmHg)	120 (112–130)	118 (110–126)	134 (125–144)	<0.001
Resting heart rate (/min)	70 (64–77)	70 (64–77)	72 (64–79)	0.002
BMI (kg/m^2^)	23.9 (21.9–26.1)	23.8 (21.7–26.0)	24.5 (22.8–26.5)	0.001
LDL (mg/dL)	118 (98–141)	117 (97–139)	126 (104–150)	<0.001
Glucose (mg/dL)	89 (80–98)	88 (80–96)	96 (85–107)	<0.001
Smoking	186 (9.6)	149 (9.4)	37 (10.7)	0.462
Physical activity *	974 (50.30)	805 (50.7)	169 (48.7)	0.509
Hypertension	234 (12.09)	158 (9.9)	76 (21.9)	<0.001
Hyperlipidemia	98 (5.06)	72 (4.5)	26 (7.5)	0.023

Normal baPWV was defined as baPWV < 1400 cm/s. High baPWV was defined as baPWV ≥ 1400 cm/s. * In addition, the participants were defined as physically active if they engaged in more than 150 min of moderate-intensity exercise in a week. Abbreviations: baPWV, brachial-ankle pulse wave velocity; BMI, body mass index; LDL, low-density lipoprotein cholesterol.

**Table 2:** Trend analysis of depressive and anxiety symptoms and poor quality of life in normal and high baPWV groups.

	Normal baPWV	High baPWV	*p* Value	*p* for Trend
Depressive symptoms	72 (4.5)	26 (7.5)	0.023	0.023
Anxiety symptoms	155 (10.1)	35 (10.5)	0.801	0.802
Poor QOL	42 (2.7)	10 (3.0)	0.755	0.755

Normal baPWV was defined as baPWV < 1400 cm/s. High baPWV was defined as baPWV ≥ 1400 cm/s. Depressive symptoms were defined as beck’s depressive symptoms index ≥ 20. Anxiety symptoms were defined as beck’s anxiety symptoms index ≥ 16. Poor quality of life is defined as WHOQOL-Bref ≤ 59. Abbreviations: baPWV, brachial-ankle pulse wave velocity; QOL, quality of life; WHOQOL-Bref, World Health Organization’s Quality of Life Questionnaire.

**Table 3:** Multivariable analysis to assess the association between baPWV and depressive and anxiety symptoms and quality of life.

	Depressive Symptoms				Anxiety Symptoms			Quality of Life		
		Odds Ratio	Confidence Interval	*p* Value	Odds Ratio	Confidence Interval	*p* Value	Odds Ratio	Confidence Interval	*p* Value
High baPWV baPWV ≥ 1400 (cm/s)	Crude	1.707	1.073–2.715	0.024	1.043	0.712–1.528	0.83	1.09	0.542–2.191	0.809
	Model1	1.675	1.001–2.803	0.049	1.127	0.743–1.708	0.573	0.924	0.436–1.961	0.924
	Model2	2.003	1.120–3.581	0.019	1.328	0.838–2.105	0.227	1.201	0.527–2.738	0.664
	Model3	2.000	1.102–3.628	0.023	1.431	0.892–2.294	0.137	1.179	0.507–2.738	0.703
	Model4	1.920	1.062–3.472	0.031	1.389	0.866–2.225	0.173	1.154	0.499–2.670	0.738
	IPTW	2.637	1.219–5.704	0.014	1.823	0.988–3.364	0.055	2.259	0.948–5.384	0.066

Depressive symptoms were defined as Beck’s depressive symptoms index ≥ 20. Anxiety symptoms were defined as Beck’s anxiety symptoms index ≥ 16. Poor quality of life is defined as WHOQOL-Bref ≤ 59. Model 1 was adjusted for age and sex. Model 2 was adjusted for anthropometric measurement (systolic blood pressure, heart rate, BMI) in addition to Model 1. Model 3 was adjusted for underlying comorbidities and laboratory values (hypertension, hyperlipidemia, serum LDL, glucose) in addition to Model 2. Model 4 was adjusted for lifestyle factors, such as smoking and physical activity, in addition to Model 3. Inverse probability of treatment weighting (IPTW) was performed using Model 4. Abbreviations: ba PWV, brachial-ankle pulse wave velocity; QOL, quality of life; WHOQOL-Bref, World Health Organization’s Quality of Life Questionnaire.

**Table 4:** Multivariable analysis to assess the association between baPWV and depressive and anxiety symptoms and quality of life according to sex.

			Depressive Symptoms			Anxiety Symptoms			QOL		
			OR	95% CI	*p* Value	OR	95% CI	*p* Value	OR	95% CI	*p* Value
High baPWV baPWV ≥ 1400 (cm/s)	Men	Crude	1.821	0.871–3.807	0.111	1.549	0.752–3.187	0.235	0.681	0.231–2.007	0.487
		Model1	1.23	0.553–2.734	0.612	1.002	0.461–2.180	0.996	0.518	0.165–1.624	0.259
		Model2	1.58	0.651–3.834	0.312	1.335	0.560–3.182	0.515	0.566	0.167–1.926	0.363
		Model3	1.614	0.658–3.958	0.295	1.614	0.635–3.958	0.295	0.468	0.119–1.843	0.278
		Model4	1.529	0.626–3.736	0.351	1.529	0.626–3.736	0.351	0.451	0.115–1.762	0.252
		IPTW	2.497	1.004–6.207	0.049	1.694	0.606–4.736	0.315	0.970	0.242–3.918	0.970
	Women	Crude	1.792	0.979–3.281	0.059	1.23	0.744–1.947	0.451	1.818	0.809–4.085	0.148
		Model1	2.05	1.046–4.015	0.036	1.24	0.741–2.077	0.413	1.519	0.634–3.640	0.348
		Model2	2.239	1.034–4.847	0.041	1.452	0.809–2.608	0.211	2.409	0.894–6.492	0.082
		Model3	2.189	0.976–4.908	0.057	1.728	0.916–3.259	0.091	2.889	0.898–9.294	0.075
		Model4	2.064	0.918–4.639	0.08	1.645	0.870–3.110	0.126	2.73	0.849–8.781	0.092
		IPTW	3.003	0.989–9.120	0.052	2.148	1.000–4.612	0.050	4.561	1.465–14.199	0.009

Depressive symptoms were defined as Beck’s depressive symptoms index ≥ 20. Anxiety symptoms were defined as Beck’s anxiety symptoms index ≥ 16. Poor quality of life is defined as WHOQOL-Bref ≤ 59. Model 1 was adjusted for age and sex. Model 2 was adjusted for anthropometric measurement (systolic blood pressure, heart rate, BMI) in addition to Model 1. Model 3 was adjusted for underlying comorbidities and laboratory values (hypertension, hyperlipidemia, serum LDL, glucose) in addition to Model 2. Model 4 was adjusted for lifestyle factors, such as smoking and physical activity, in addition to Model 3. Inverse probability of treatment weighting (IPTW) was performed using Model 4. Abbreviations: baPWV, brachial-ankle pulse wave velocity; QOL, quality of life; WHOQOL-Bref, World Health Organization’s Quality of Life Questionnaire.

## Data Availability

Unavailable because the initial agreement form signed by study participants stated that the participants’ data will not be shared.

## Supplementary Materials

The following supporting information can be downloaded at: https://www.mdpi.com/article/10.3390/medicina59030477/s1.
