# Supplementary material for: A Cross-Sectional Investigation of the Association between Arterial Stiffness and Depressive Symptoms, Anxiety Symptoms, and Quality of Life
Source: Medicina (Kaunas). 2023 Feb 28;59(3):477. doi: 10.3390/medicina59030477 (PMC10057713; doi:10.3390/medicina59030477)
Supplement: Supplementary file 1 [file medicina-59-00477-s001.zip › medicina-2233363-supplementary.pdf]

Supplemental Table S1. Multivariable analysis to assess the association between baPWV and depressive, anxiety symptoms and quality of life.

			Depressive Symptoms			Anxiety Symptoms			QOL		
			**OR**	**95%CI**	***p* Value**	**OR**	**95%CI**	***p* Value**	**OR**	**95%CI**	***p* Value**
High baPWV	Age≥50 (n=835)	Crude	1.356	0.767-2.399	0.295	1.048	0.675-1.625	0.835	0.669	0.281-1.591	0.363
		Model1	1.368	0.759-2.464	0.297	1.266	0.802-1.998	0.312	0.748	0.307-1.832	0.522
		Model2	1.467	0.751-2.865	0.262	1.367	0.812-2.304	0.24	1.194	0.441-3.232	0.727
		Model3	1.48	0.748-2.929	0.26	1.415	0.800-2.501	0.233	1.024	0.340-3.085	0.966
		Model4	1.438	0.726-2.846	0.297	1.419	0.801-2.514	0.231	1.021	0.339-3.074	0.971
		IPTW	1.685	0.843-3.370	0.14	1.221	0.701-2.128	0.481	1.671	0.605-4.618	0.322
	Age<50 (n=1101)	Crude	1.875	0.716-4.909	0.201	1.214	0.591-2.495	0.597	2.879	1.075-7.715	0.035
		Model1	2.727	0.976-7.622	0.056	1.338	0.634-2.824	0.446	2.055	0.729-5.792	0.03
		Model2	4.097	1.261-13.314	0.019	1.666	0.730-3.803	0.225	2.163	0.666-7.027	0.2
		Model3	4.143	1.220-14.069	0.023	1.64	0.664-4.053	0.284	2.258	0.604-8.448	0.226
		Model4	3.364	0.980-11.549	0.054	1.428	0.571-3.571	0.446	1.88	0.488-7.274	0.361
		IPTW	4.959	0.882-28.529	0.073	2.153	0.450-10.311	0.337	2.153	0.450-10.311	0.337

Depressive symptoms were defined as beck's depressive symptoms index ≥20
Anxiety symptoms were defined as beck's anxiety symptoms index ≥16
Poor quality of life is defined as WHOQOL-Bref≤59
Model 1 was adjusted for age and sex.
Model 2 was adjusted for anthropometric measurement (systolic blood pressure, heart rate, BMI) in addition to model 1.
Model 3 was adjusted for underlying comorbidities and laboratory values (hypertension, hyperlipidemia, serum LDL, glucose) in addition to model 2.
Model 4 was adjusted for lifestyle factors, such as smoking and physical activity, in addition to Model 3.
Abbreviations: baPWV, brachial-ankle pulse wave velocity; QOL, quality of life; WHOQOL-Bref, World Health Organization's Quality of Life Questionnaire.
